# The evolution of methods for establishing evolutionary timescales

**DOI:** 10.1098/rstb.2016.0020

**Published:** 2016-07-19

**Authors:** Philip C. J. Donoghue, Ziheng Yang

**Affiliations:** 1 School of Earth Sciences, University of Bristol, Life Sciences Building, Tyndall Avenue, Bristol BS8 1TQ, UK; 2 Department of Genetics, Evolution and Environment, University College London, Gower Street, London WC1E 6BT, UK

**Keywords:** molecular clock dating, divergence times, Bayesian inference, fossils, node-calibrations, tip-calibrations

## Abstract

The fossil record is well known to be incomplete. Read literally, it provides a distorted view of the history of species divergence and extinction, because different species have different propensities to fossilize, the amount of rock fluctuates over geological timescales, as does the nature of the environments that it preserves. Even so, patterns in the fossil evidence allow us to assess the incompleteness of the fossil record. While the molecular clock can be used to extend the time estimates from fossil species to lineages not represented in the fossil record, fossils are the only source of information concerning absolute (geological) times in molecular dating analysis. We review different ways of incorporating fossil evidence in modern clock dating analyses, including node-calibrations where lineage divergence times are constrained using probability densities and tip-calibrations where fossil species at the tips of the tree are assigned dates from dated rock strata. While node-calibrations are often constructed by a crude assessment of the fossil evidence and thus involves arbitrariness, tip-calibrations may be too sensitive to the prior on divergence times or the branching process and influenced unduly affected by well-known problems of morphological character evolution, such as environmental influence on morphological phenotypes, correlation among traits, and convergent evolution in disparate species. We discuss the utility of time information from fossils in phylogeny estimation and the search for ancestors in the fossil record.

This article is part of the themed issue ‘Dating species divergences using rocks and clocks’.

## 1. Introduction

Approaches to inference of evolutionary history have a patchy record, punctuated as much by the discovery of new types of data, as by changing philosophies in which data are interpreted. Early phylogenies were based on comparative analysis of living species, whether based on embryology or anatomy, and guided by perceived laws of ‘natural affinity’, increasing complexity, or divinity [1]. Fossil species played a secondary role, providing evidence for the gradual or episodic evolution of organisms, from primitive to advanced. At the same time, perceptions of the extent of the evolutionary history of Life on Earth have been transformed, from the several million years that Darwin and the majority of his contemporaries would have perceived [2], through to the tens, hundreds and, ultimately, thousands of millions of years that were revealed by radiometric dating [3].

**Figure 1.**
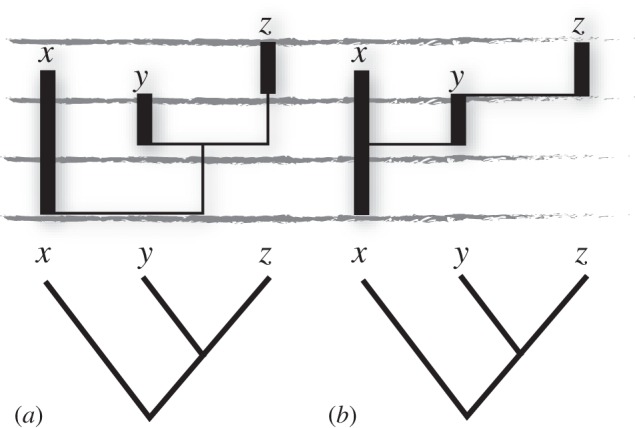
The relationship between cladograms, which consider only relative relationships, and phylogenies, which can represent absolute relationships. Both (*a*,*b*) are compatible with the same hypothesis of cladistic relationships; however, (*b*) represents a hypothesis of budding ancestry between (*x*,*y*), and anagenesis between (*y*,*z*). Phylogeny (*a*) implies gaps (represented by thin vertical lines subtending the thick vertical bars which reflect the stratigraphic ranges of taxa *x*–*z*) in the fossil record to accommodate the sister group relationship between lineages *x* and *y* + *z*, and between *y* and *z*. Meanwhile phylogeny (*b*) does not imply any gaps in the fossil record.

Calibrating the Tree of Life to geological time has traditionally been the preserve of palaeontologists, initially placing more significance on the stratigraphic distribution of fossil species than on their place within a grand Tree of Life. The goal of a universal phylogeny was unrealistic before the discovery of universal genes, and palaeontologists in the New Synthesis had a microevolutionary focus, to infer evolutionary rates on timescales that would blend with studies of living species [4]. Detailed stratigraphic analysis has demonstrated that for some fossil groups, such as the unicellular foraminifera, ancestor–descendent relationships can be discerned among morphospecies, as one can be traced morphing gradually into another based on morphological characters (e.g. [5]). However, this ‘stratophenetic’ approach is only suitable for groups with a rich fossil record (e.g. [6,7]). Indeed, even for those groups with the most complete fossil record, attempts to reconstruct time-calibrated phylogenies are confounded by gaps and the heterogeneous structure of the rock record [8]. For most groups, stratigraphic samples are smaller, there are stronger ecological and, therefore, geographical controls on distribution, and fossilization is less common. Combined with the non-uniform preservation of environments in the rock record [9], the fossil records of most groups are too non-uniform for stratophenetic approaches, and relative (rather than absolute ancestor–descendent) evolutionary relationships are the most that can be achieved with any degree of certainty. This fact is borne out by a general lack of correlation between the order of stratigraphic appearance and phylogenetic branching among fossil species, since sibling-lineages should exhibit contemporaneous first fossil occurrences [10,11] (figure 1*a*). Theoretical objections have been raised against the practice of identifying ancestors among fossil taxa since species do not beget other species but, rather, they emerge through differentiation among populations [12] and, thus, to identify an ancestral species is a category error—it effectively identifies the species as paraphyletic, only components among which can be considered as potential ancestors. And hypotheses of ancestry are also problematic because they rely upon negative evidence, i.e. the fact that putative ancestors preserve only plesiomorphic characters and lack autapomorphies that might distinguish them as distinct lineages [13,14].

Thus, for many palaeontologists, stratigraphic time has little role in phylogeny estimation, except in providing minimum ages to calibrate morphology-based cladograms to time, or in discriminating among multiple trees of equal likelihood or parsimony [14]. This is not to say that ancestors do not exist in the fossil record [15–17] and failure to accommodate fossil species will result in the perception of gaps in the fossil record where there are none [18–20]. This occurs because ancestral taxa are misrepresented as sibling-lineages of their descendants, resulting in the perception of a gap in the fossil record of the descendent, given the expectation that sibling-lineages diverge contemporaneously from their last shared ancestor [10] (figure 1). Parsimony and likelihood-based ‘stratocladistic’ methods have been developed that attempt to minimize perceived gaps in the fossil record, not least through the recognition of ancestor–descendent relationships among fossil morphospecies [21,22]. However, notwithstanding the likelihood of ancestors among the fossil species, the veracity of attempts to identify specific ancestral morphospecies remains questionable [13,23]. As such, stratocladistic methods may serve to conceal embarrassing gaps in the fossil record, making it appear a much better archive of evolutionary history than it really is.

Clearly, for the majority of clades, the fossil record alone is not sufficient to establish anything more than a minimum estimate for the age of living and fossil clades; only the molecular clock provides a means of approaching a true evolutionary timescale. Below, we review the history of development of molecular clock methodology, and the use of fossils and morphological data to calibrate the molecular clock. We discuss the potentials and challenges of modern Bayesian dating methods, which attempt to integrate different sources of information in one combined analysis, such as distance information in genetic sequences and time information in the fossil record. We highlight challenges confronting the latest methodological developments in divergence time estimation and show that these retread long-standing debates associated with phylogeny and timescale estimation in palaeontology, from which insights might be gained for the future development of molecular clock methodology.

## 2. The origin and early evolution of the molecular clock

The molecular clock hypothesis was conceived from the observation that the differences between homologous amino acid sequences from different mammal species is roughly proportional to their time of divergence [24,25]. If the time of divergence between any pair of species is known, such as based on the oldest fossil record from one of the pair of lineages, then the rate of molecular evolution can be inferred and used to date the timing of divergence between other species pairs. The molecular clock was widely employed to date species divergences in the 1990s when molecular sequence data first became available for diverse lineages. However, many of those early analyses produced extremely ancient divergence time estimates, such as a Mesoproterozoic origin of bilaterian animals [26], a Cryogenian origin of land plants [27], a deep Jurassic origin of flowering plants [28] and a deep Cretaceous origin of the ordinal level crown groups of birds and eutherian mammals [29]. These estimates challenged not only the veracity of the fossil record as an archive of evolutionary history, but also macroevolutionary hypotheses that had been based on fossil data, such as the end-Cretaceous mass extinction and its role in shaping modern biodiversity, which had effectively become philosophies in which those fossil data were interpreted.

Given the general acceptance among palaeontologists of the incompleteness of the fossil record, it might be imagined that their discipline would embrace the molecular clock hypothesis with giddy enthusiasm and, indeed, palaeontologists were among its earliest adopters (e.g. [30,31]). However, the large mismatch between the early molecular date estimates and the fossil evidence of clade ages led many palaeontologists to reject these molecular estimates out of hand [26,28,32].

There are many reasons to expect clade ages to be older than their oldest fossil representatives. First, evidence of lineage separation cannot be manifest in the fossil record until one or both of the descendent species have acquired distinguishing anatomical characteristics that have the potential to be fossilized. By contrast, molecules evolve independently as lineages diverge. The difference between the time of lineage divergence and the age of the oldest fossil species can be even greater due to uneven species distributions and variation in the preservation of the sediments (facies) in those environments. For example, the oldest definitive records of terrestrial plant and animal lineages are approximately equivalent [33]. However, rather than an explosive radiation of terrestrial organisms, this correlated appearance more likely reflects the dearth of terrestrial sediments from which to sample fossils immediately prior to the middle Silurian [34]. There is also the challenge of correctly assigning derived species, because early diverging lineages are difficult to distinguish from their ancestors on anatomical grounds, and when derived characters are few in number it can be difficult to discern whether such characters are genuinely derived or evolved ancestrally before being lost in one of the derived lineages. This is complicated further by the incompleteness of fossil preservation, since corroborative anatomical characters are needed to distinguish between shared derived characters and convergences or parallelisms. For instance, the earliest fossil chondrichthyans are distinguished on the presence of vascular canals associated with the ‘neck’ of the attachment process of their scales. However, these fossils are limited to isolated scales [35,36]. How certain can we be that scale neck canals evolved only once, or that they are a primitive chondrichthyan character, or that they are not a shared primitive character lost in osteichthyans? Corroborative anatomical evidence would be useful to determine that these microremains belonged to a jawed vertebrate.

**Figure 2.**
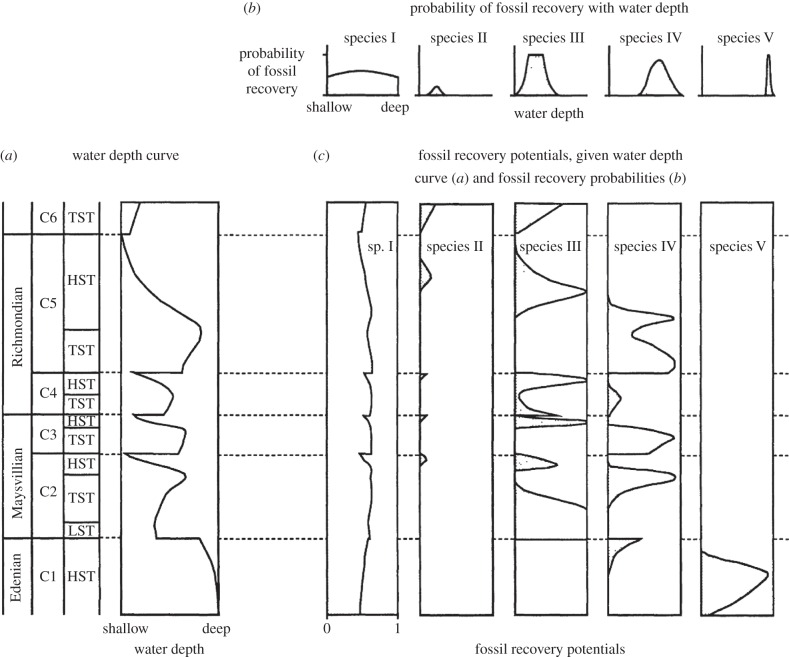
The predictable nature of fossil occurrences. Fossil stratigraphic occurrences are distinctly non-random, determined by the environmental controls on the distribution of the living organism and secular variation in the preservation of the environment in which the organism lived and died. Thus, fossil occurrences can be predicted based on knowledge of environmental limits of their distribution and characterization of environments and how they vary through stratigraphic sections and their global composites. (*a*) Inferred variance in water depth through a stratigraphic section; (*b*) probability of recovering a fossil based on its water depth tolerance and (*c*) fossil recovery potential given (*a*,*b*). Reproduced from Marshall [40] with the permission of the author and publisher.

In combination, these factors result in significant differences between the timing of divergence and the age of the oldest fossil, but they cannot account for the scale of the mismatch implied by many early molecular clock studies. This is because gaps in the fossil record are largely predictable, based on the quality of the fossil record, how it varies between groups, and how fossil species' stratigraphic ranges may be influenced by secular variation in the preservation of facies in the rock record. For instance, palaeobiologists conduct gruesome decay experiments to discern the relative preservation of anatomical characters, and of taxa [37]. Further, knowledge of the sedimentary facies associations of fossil species can be exploited to predict probabilistically their occurrence through stratigraphic sequences [38,39] (figure 2). With suitable taphonomic controls [41], unfulfilled predictions of fossil stratigraphic occurrences can be interpreted as evidence for the absence of those fossil species in space and time.

Thus, despite the incomplete and non-uniform nature of the fossil record, it is safe to conclude that many of the great conflicts between the molecular time estimates and the fossil record are to a large extent due to the many limitations of the early clock dating studies [42]. These include the incorrect assumption of the strict clock or inadequate accommodation of the violation of the clock when dating deep divergences, the use of calibrations that ignore uncertainties in fossil evidence, as well as the use of secondary calibrations and substitution rates (that is, node ages and substitution rates estimated in previous molecular clock dating studies). While methods that effectively assume a strict clock continue to be employed and developed [43,44], producing unreasonably ancient divergence time estimates [45], they are hard to justify given the overwhelming evidence for violation of the clock among distant species. Nevertheless, in generating controversy, the early dating analyses were influential in shaking evolutionary biologists out of the view that evolutionary timescales could be read from the fossil record, and forced palaeontologists to review its utility and limits. They also prompted communities to reconsider their cherished hypotheses, such as the role of mass extinctions in shaping modern biodiversity. However, these timescales have not withstood the test of time, and they are now considered only in terms of their historical value in charting the history of development of this scientific method.

## 3. The modern molecular clock and the challenge of calibrating it to geological time

Mismatches between molecular clock estimates and clade ages based on the oldest fossil occurrences now rarely occur on the scale that they did in the 1990s. The main reason for this change is the development of analytical methods that can accommodate the violation of the clock as well as uncertainties in fossil calibrations. Although the earliest relaxed-clock methods were developed within a likelihood framework [46,47], more recent advances have been implemented within a Bayesian framework [48–54].

This is a straightforward application of Bayesian inference, in which the parameters of interest are the species divergence times (***t***), the molecular evolutionary rates for branches on the tree (***r***), the parameters in the substitution model and in the prior (*θ*), while the data are the sequence alignments at multiple gene loci (*S*). A Markov chain Monte Carlo algorithm is used to sample from the joint posterior
3.1
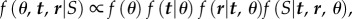

where *f*(*θ*) is the prior for parameters, *f*(***t***|*θ*) the time prior, specified using a branching process such as the birth–death sampling model [55,56], *f*(***r***|***t***, *θ*) is the prior for the rates, and *f*(*S*|***t***, ***r***) is the likelihood for the sequence data. The times are shared among the multiple loci. In this formulation, the joint prior density of divergence times, *f*(***t***|*θ*), incorporates fossil calibration information whenever it is available, with the distribution of ages of other nodes supplanted by the branching process such as the birth–death sampling model.

It may be important to note that the likelihood for the sequence data, *f*(*S*|***t***, ***r***, *θ*), depends on the branch length, which is the expected number of changes on each branch, and is the product of the time duration for the branch and the rate for the branch. In other words, times and rates are confounded. A consequence of this confounding effect is that even if a huge amount of sequence data is analysed, the posterior of times and rates will remain sensitive to the prior on times and prior on rates [52,57,58]. Thus, having accurate fossil or temporal constraints is always important to a molecular clock dating analysis.

In this article, we focus on the time prior *f*(***t***|*θ*). Two principal approaches to calibrations have been implemented, sometimes referred to as node-dating and tip-dating; since both approaches to calibration are used to date nodes, it is preferable to refer to them as node-calibration and tip-calibration, respectively [59]. Below, we describe the different approaches and the motivations underpinning their development, before considering their relative merits and areas for their future development.

### (a) Strategies to derive node-calibrations

Interpreting the fossil evidence to construct calibrations for molecular clock dating is a challenging task. The fossil record can directly inform the minimum ages of clades based on the age of their oldest fossil representative [60,61]. Establishing a maximum constraint is far more problematic since it relies on the interpretation of negative evidence—the absence of fossil evidence for a clade may be due to the vagaries of fossilization rather than simply because the clade had yet to evolve. Several pragmatic solutions have been proposed to establish maximum age constraints on clade ages. The simplest approach is to use a parametric distribution (such as the gamma, lognormal or the truncated Gaussian) to express the probability of the true divergence time relative to the minimum fossil-based age-constraint [52,62]. Without a statistical analysis of the fossil data, this approach inevitably involves some arbitrariness. While the precise parametric forms for node-calibrations (such as the gamma versus a pair of minimum and maximum bounds) were found to be unimportant in some studies (e.g. [63]), whether soft maximum bounds were included in a dating analysis has been found to have a dramatic effect on the posterior time estimates [64,65].

Another approach, phylogenetic bracketing, exploits the predictive nature of fossil occurrences based on their sedimentary facies associations and stratigraphic facies variation, to interpret the absence of fossil representatives of the clade ingroup [66,67]. It is important, however, that outgroup taphonomic control species are used, which have the same ecological and preservational characteristics of the ingroup, to discriminate absences that occur simply because the conditions required for fossilization were not met, or else because older sediments do not represent the environments that the organisms inhabited. Confidence intervals can be calculated to infer the true stratigraphic range of calibrating fossils, which correspond to the minimum bounds for the true age of lineage divergence [40,68,69]. However, their utility in inferring the age of clades or lineage divergences is limited [70], and it is not useful for interpreting the fossil record of species known from single individuals or single stratigraphic levels.

Node-calibrations typically do not satisfy the requirement that ancestral species should be older than their descendants. If for no other reason, this occurs because the uncertainties associated with the age-priors on ancestor–descendent nodes frequently overlap. Specifying multi-dimensional priors in Bayesian inference is in general a challenging task, and in the case of specifying calibrations for multiple nodes on the tree, with some nodes being ancestral to others, this task is daunting without statistical analysis of the fossil data, as in [63,71]. Current Bayesian programs multiply the densities for the calibration nodes first, and then when the program is run, node ages that violate the biological constraint are disallowed, effectively truncating the joint prior distribution of the ages for all calibration nodes, e.g. [52]. Thus, the effective prior used by the computer program may be quite different from the user-specified priors [64,72]. Different programs may use different strategies to apply this truncation, leading to different effective priors even if the user initially specifies exactly the same calibrations [64,65,72]. Therefore, it is important that the effective priors are scrutinized (by running the analysis without sequence data) to ensure that they are compatible with the palaeontological and phylogenetic evidence on which the specified node-calibrations were originally based.

The most sophisticated among this class of methods is the probabilistic modelling of fossil preservation and discovery, and statistical analysis of the absence and presence data of fossil species in different rock strata by Tavare *et al*. [71] and Wilkinson *et al*. [63]. Bayesian analysis of the fossil data alone (the presence and absence of fossil species in the different rock strata) produces a posterior distribution of node ages, which can then be used as the prior of times in the subsequent molecular dating analysis using the sequence data. This is based on a branching-process model that describes speciation and extinction, as well as fossil preservation and discovery, assigning priors on the speciation rate, extinction rate and sampling intensity. This approach is attractive as it makes use of information in all the pertinent fossils, in contrast to other node-calibration methods that use only the oldest fossil that constrains directly the age of the extant clade.

### (b) Integrative analysis of fossil and sequence data

The fossilized birth–death (FBD) model [73] is a similar to the statistical methods developed by Tavaré and co-workers [63,71], except that it analyses the fossil data jointly with the molecular sequence data. It attempts to describe both the distribution of fossils and the lineage divergence times within a clade based on an integrated diversification–fossilization model. In its original incarnation [73], fossil species are assigned to clades with varying degrees of precision, and clade ages are inferred in a conventional Bayesian molecular clock analysis. The model requires only priors on the speciation and extinction rates, the fossil recovery rate and the proportion of extant species sampled. It assumes constant speciation and extinction rates, initiating on a single lineage and identifying fossil species according to a Poisson process, and extant species at a given probability. This tree is stripped of unsampled extant and extinct species, yielding the reconstructed phylogeny of living and fossil species. However, the FBD can integrate over the uncertainty of the phylogenetic position of the fossil species and the timing of their divergence from extant lineages, including the possibility that fossil species diverged at a time equal to their age, i.e. that the fossil species is effectively ancestral to the extant lineage. This unresolved FBD model does not use any information from morphological characters or measurements for the fossil species other than its geologic age—represented by a time point sampled from its stratigraphic range and attendant age uncertainty [73]. This has both positive and negative implications. In that the model requires only qualified phylogenetic affinity and fossil age, the unresolved FBD facilitates the integration of all pertinent fossil information. However, the model always requires certainty in the phylogenetic affinity of fossil species among their extant relatives, and the impact of phylogenetic precision will propagate to the resulting divergence time estimates. Since the FBD integrates over uncertainty associated with the phylogenetic affinity and the timing of lineage divergence of extinct species, it provides insight only into the antiquity of extant clades. Nevertheless, the FBD model is an attractive objective method for deriving estimates of clade ages from phylogenetic trees and palaeontological data. Importantly, it gets around the inconsistency between the specified and effectives time priors that derive from conventional node-calibration.

### (c) Tip-calibration and the joint analysis of molecular and morphological data

**Figure 3.**
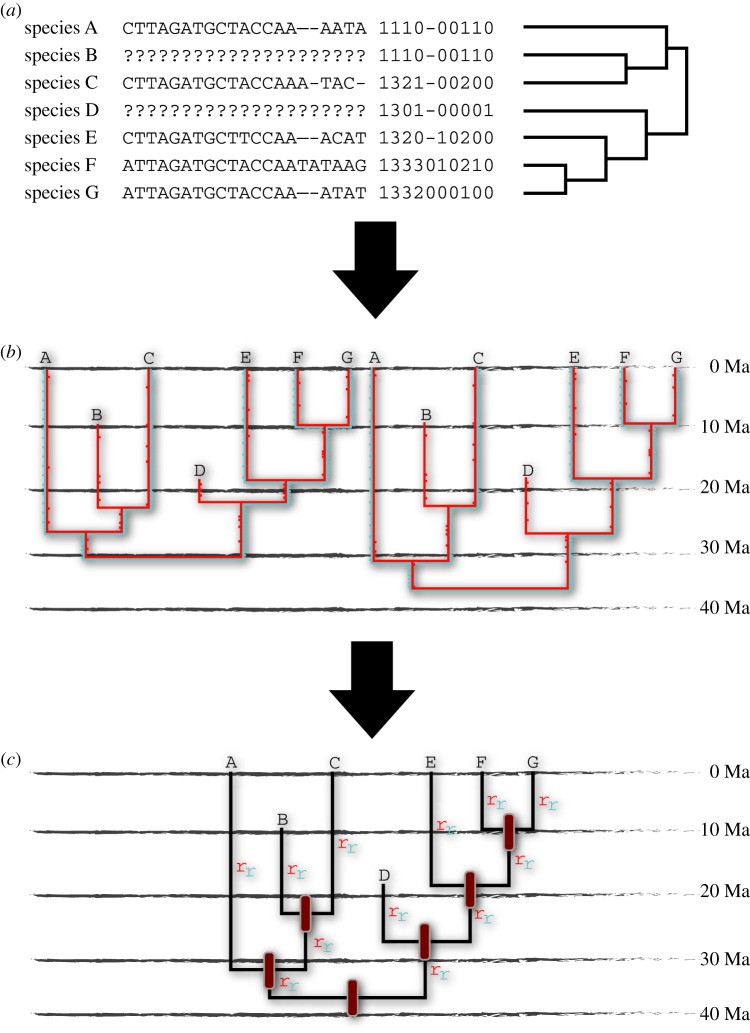
Tip-calibration relies upon a molecular sequence alignment from living species, a morphological character set for living and fossil species, and a prior topology (*a*); total-evidence dating co-estimates topology and timescale. Branch lengths are estimated in a Bayesian MCMC approach based on both data types for living lineages and based on morphological data alone for the extinct lineages; these are calibrated to time based on the age of the fossil species (*b*). The divergence time estimates and inferred rates of molecular and morphological evolution are based on a consensus of the MCMC analysis (*c*). (Online version in colour.)

The desire to overcome inconsistency between specified and effective node-calibrations is one of the motivations behind the development of alternative approaches to calibrating molecular clocks through the integration of morphological data and tip-calibration methods that include fossil species as terminal taxa among their extant relatives [16,74] (figure 3). Tip-calibration is often considered synonymous with the so-called total-evidence dating approach that also facilitates the simultaneous estimation of time and topology [16]. However, because these two approaches can be employed separately, we will first consider tip-calibration before going on to appraise total-evidence dating.

Tip-calibration is achieved by analysing morphological data for both living and fossil species under a model of morphological character evolution and molecular sequence data for living species [16,74]. The formulation is similar to equation (3.1), with the differences that we also have morphological data for fossil and modern species (*M*), besides the sequence data for modern species (*S*) and that there are two sets of rates, ***r***_S_ for sequences and ***r***_M_ for morphology. The joint posterior is then
3.2
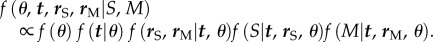

Here *f*(***r***_S_, ***r****_X_*|***t***, *θ*) is the prior for the rates, while *f*(*M*|***t***, ***r***_M_) is the likelihood for morphological data. The times are shared between the molecular and morphological data. In the simple case, similar rate-drift models (such as the geometric Brownian motion model) can be used to describe variable morphological rates among lineages as in the case for sequences.

Tip-calibration is theoretically attractive as it involves the simultaneous and, thus, coherent analysis of morphological data for both fossil and extant species and sequence data from modern species. It also integrates the time information from fossil species directly, rather than indirectly constraining the ages of clades, as in node-calibration (figure 3). Branch lengths of extant lineages are estimated based on morphological and molecular data while those of extinct lineages are estimated based on morphological data alone. Ultimately, these are calibrated to time based on the age of the fossil taxa, integrating stratigraphic age range and its attendant errors, as appropriate. It provides a means of estimating the ages of all clades, not merely those with living descendants. Indeed, the approach can be applied to fossil data alone, without sequence data, as has been done to date the timing of divergence of avian and non-avian dinosaurs [75,76], crown-Aves [77] and placental mammals [78].

### (d) Total-evidence dating

The importance of fossil species in informing the relationships of extant species has long been emphasized [79,80]. The introduction of tip-calibration and the morphological clock has facilitated the development of methods for the co-estimation of phylogenetic relationships and their absolute timescales [16,74]. Thus, it is possible to integrate the phylogenetic uncertainty of living and fossil taxa in a manner that is very difficult to accommodate using node-calibrations that must make at least minimal assumptions concerning the phylogenetic relationships of living lineages and the place of fossil taxa among them. Furthermore, implementations of the total-evidence dating approach make it possible to use the age of fossil species to inform their phylogenetic position, following the expectation that fossil representatives of early diverging lineages will be older than fossil representatives of lineages that diverge later in time. Indeed, even increasing uncertainty in the age of a fossil can lead to a changed phylogenetic hypothesis using total-evidence dating [59].

Extensions of the total-evidence dating method allow fossil species to be accommodated as direct ancestors of extant species, as well as the implementation of more flexible branching-process models such as the FBD model as well as those that allow diversified sampling of extant species [81,82]. This has the impact of diminishing uncertainty in the age of clades, overcoming the tendency of tip-calibration to yield ancient clade age estimates. It achieves this directly, by accommodating ancestral species that, were they identified as distinct lineages, would inflate branch lengths, the age of clades and, consequently, the extent of perceived gaps in the fossil record [81].

## 4. Discussion

Molecular clock methodology is currently undergoing such rapid development that it can be difficult to discriminate which methodological approach best suits the problem at hand. Indeed, the introduction of tip-calibration and the co-estimation of phylogenies and their timescales could be interpreted as replacements for conventional node-calibration and sequential analysis of phylogeny and timescales. There are strong arguments that favour the integration of all relevant lines of evidence, and their simultaneous analysis to derive time-calibrated phylogenies. Not least, these include the unchallengeable view that morphological and molecular data, living and fossil species, are all a consequence of the same evolutionary process. Meanwhile, sequential analysis of a phylogeny of extant taxa usually employs only molecular data; node-calibrations for the component clades are derived based on the phylogenetic position of fossil species within this scheme, based on morphological data. These are transformed in the assembly of the joint time prior and integrated into a molecular clock analysis that otherwise uses only molecular sequence data and the original molecular phylogeny. Implicitly, at least, this approach makes the assumption of independence among the different models and data [82]. However, despite their theoretical appeal, tip-calibration and total-evidence dating methods face a number of challenges, and exhibit strong parallels to the challenges that have long confronted the construction of time-calibrated phylogenies in palaeontology [83–85]. Many of those challenges have not yet been systematically explored.

### (a) Tip-calibrations are very sensitive to the branching process or the prior for times

**Figure 4.**
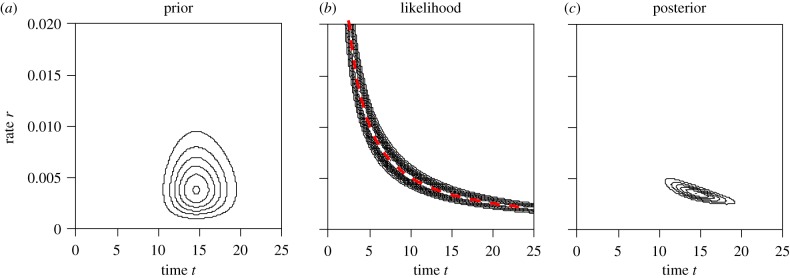
(*a*) Estimation of the absolute time (*t*) and rate (*r*) using the human and orangutan 12S rRNA genes from the mitochondrial genome. The data are summarized as *x* = 90 differences out of *n* = 948 aligned sites. The likelihood, calculated under the JC69 substitution model [86], depends on the distance *d* = 2*tr* only, but not *t* and *r* individually. The maximum-likelihood estimate of *d* under the JC69 model is 0.1015, with the 95% confidence (likelihood) interval to be (0.0817, 0.1245). All points on the red dashed line in (*b*) correspond to the same likelihood value and are maximum-likelihood estimates of *t* and *r*. To generate the posterior of *t* and *r*, we assign the prior *t* ∼ *G*(40, 40/15), with the prior mean to be 15 Myr and the 95% equal-tail interval to be (10.7, 20.0) Myr, and the rate prior *r* ∼ *G*(4, 800), with the mean to be 0.005 substitution per million years and the 95% interval to be (0.14, 1.10). (*c*) Relatively, our prior knowledge of the rate is less certain than that for time. Note that to obtain sensible posterior time estimates, it is important to constrain the time from both below and above in the prior (in this case, the time is weakly constrained to be in the range 10–20 Myr). (Online version in colour.)

The most serious problem facing tip-calibration may be the extreme sensitivity of the posterior time estimates to the prior of divergence times specified by the branching process. Because the sequence data provide information about distances only, resolution of the sequence distance into absolute time and rate relies entirely on the priors on time and rate (figure 4). Most tip-calibration methods require a bound or prior on the age of the root for extant species, e.g. [16], but no calibrations are applied on the ages of other internal nodes. Thus, node ages are bounded by the ages of the fossil tips, because ancestral nodes cannot be younger than their descendent fossil tips, while there is otherwise effectively no constraint on the ages of clades except for the prior on the root age. In other words, there are multiple forces pushing up the node ages, but almost no force pushing them down. It is left to the divergence time prior or the branching-process model to keep the node ages on the tree within reasonable bounds, and that may prove to be too much burden on the time prior. A dozen or so initial studies applying the total-evidence dating approach have produced ancient time estimates, older even than those derived from the use of node-calibrations [59]. This is remedied by incorporating the FBD model into total-evidence dating, replacing the original uniform tree prior and making use of morphological character data in resolving the affinity of fossil taxa [81,82]. However, details of the FBD prior, such as the assumed sampling regime, can have a strong influence on divergence time estimates [81].

For example, when species sampling is assumed to be complete or uniform in the branching process (the BDS model), Ronquist *et al*. [16] dated crown Hymenoptera to the late Carboniferous (309 Ma). Changing the prior model to account for diversified sampling dated the crown radiation to the Permo-Triassic (252 Ma), 57 million years younger [81]. Condamine *et al*. [87] provide a similar cautionary tale [87] in dating the origin of living cycads based on node-calibrations. While diversified sampling is arguably a more realistic prior model than uniform sampling, it may be naive to expect it to capture the main features of the complicated process of species sampling. Furthermore, there is very little information in the data to estimate the speciation rate, the extinction rate and the sampling rate in a birth–death sampling model when those rates are changing over time [88,89]. The sensitivity of the posterior time estimates to the prior branching-process model is troubling, but the effect is a general feature of the model formulation, not limited to particular datasets. Diversified sampling causes the tree to become ‘bush-like’, while a low fossilization rate and high extinction rate push fossils towards extinct side branches [81]. Because sequence data are not informative about those rates, the assumptions in the prior model translate directly into the posterior time estimates. Thus, we stress the importance of assessing the robustness of posterior time estimates to the prior in Bayesian relaxed-clock dating analysis, especially the prior on times (or the branching process) and the prior on substitution rates for multiple loci. In combined analysis of molecular and morphological data, it is important to assess the impact of the morphological model.

A pragmatic solution may be to combine tip and node-calibrations in the same analysis [59,90], although there are challenges to this approach. First, it would be inappropriate to use the same fossil data to inform both tip- and node-calibrations in the same analysis. Second, the prior density for divergence times under the birth–death sampling model, when some node ages are constrained by node-calibrations, may not be tractable analytically. Third, node-calibrations are contingent upon a prior hypothesis of relationships and so they are incompatible with attempts to co-estimate time and topology, as in the classic total-evidence dating approach. These concerns can, however, be overcome by not using the same fossil data to inform tip- and node-calibrations, and facilitating coestimation with a minimal backbone topology constraint compatible with the few nodes that are calibrated.

### (b) A morphological clock?

In a tip-calibrated analysis, the information about absolute divergence times ultimately comes from the fossil record and the assumed clock-like evolution of morphological characters. Thus, the many weaknesses of morphological data identified in the molecules-versus-morphology debate of the early 1990s remain important to the use of morphology to estimate branch lengths and to date molecular trees. For example, phenotypes are influenced by the environment as well as by genes, and morphological characters may undergo convergent evolution in disparate lineages. Models of morphological evolution have undergone very little development and the most widely used, *Mk* model [91], is a generalization of the JC69 model of molecular sequence evolution, the inherent assumptions underpinning which are not entirely appropriate for the analysis of morphological data, including independence among sites [59]. Furthermore, the evolution of morphological characters is not clock-like, as a rule, even among closely related species [92], and so the existence of a morphological clock, no matter how relaxed, remains questionable. The non-clock-like behaviour of morphological evolution may have far greater impact on divergence time estimation than on phylogeny reconstruction.

Finally, while it is a truism to observe that morphological clock analyses are limited by the availability of morphological data, this is usually considered in terms of fossil taxa, but living taxa are particularly poorly characterized in terms of their anatomy [93]. Missing morphological data are also non-uniformly distributed, as a consequence of the work of organ- and taxon-specialists of living organisms, and of decay in fossils where non-biomineralized tissues and organs and tissues are not preserved except in the most exceptional circumstances. This can lead to a systematic bias in phylogeny estimation where fossil species lacking derived characters (as an artefact of incomplete fossilization) are resolved as less-derived phylogenetically than they really are [83,85]. This has significant implications for molecular clock calibration in general, including the formulation of node-calibrations [84]. However, this bias probably has greatest impact on tip-calibration because of its influence on topology and branch length estimation [59].

### (c) Co-estimation versus sequential analysis of topology and time

Co-estimation of phylogenies and their timescales using tip-calibration promises to provide a basis for establishing a correct timescale for extinct, not merely extant, clades. However, in reality, this promise is not commonly realized when an appreciable number of fossil species are included since the phylogenetic positions of the fossil taxa are not usually well resolved (e.g. [16,81,94,95]). Rather, the fossil species serve largely to inform the age of living clades, as in node-calibration. This is likely because the (invariably incomplete) phenotypic character data are insufficiently informative on the phylogenetic position of the fossil species relative to the living species, the phylogenetic position of which are informed by both molecular and morphological data. Clearly, greater insight may be obtained into the age of extinct clades, and the timing and rates of character evolution, by instead dating a more fully resolved and separately justified tree within a sequential analysis of phylogeny and timescale.

Notwithstanding the merits of co-estimating time and topology, current implementations of the total-evidence dating approach use the ages of fossil species to inform their phylogenetic position (e.g. [16]). Indeed, even increasing uncertainty in the age of a fossil can lead to changes to the inferred phylogeny [59]. However, the underlying expectation, that the stratigraphic order of fossil species reflects their phylogenetic branching order, is contingent on the completeness of the fossil record of a clade [96] or at least its sampling within an analysis. Synoptic analyses have demonstrated significant inconsistency in stratigraphic and phylogenetic branching order, even in groups that are considered to have a rich fossil record [11,97]. Development of methods for estimating topology using fossil age information could benefit from the long-standing palaeontological debate on the topic. Indeed, methods have already been developed to rationalize phenotypic character evolution, topology and the stratigraphic range of fossil species (e.g. [22,98,99]). However, such methods must be developed to accommodate controls on the distribution of fossil species, which include their relative fossilization potential, the impact of non-uniform preservation of phenotypic characters [83,84], as well as secular variation in the sedimentary facies preserved in the stratigraphic record [9,41]. The beginnings of such an approach, controlling for the non-uniform nature of fossil distribution, are present in the FBD model [73,81]. However, codifying these priors for the model will be a challenge, especially for analyses that must consider the global palaeontological and, therefore, stratigraphic record.

### (d) In search of ancestors

Palaeontologists have long debated the possibility of inferring direct ancestors, as well as the merits and demerits of representing such absolute relationships among phylogenetic hypotheses (e.g. [100–103]). As we have discussed, there can be little doubt that ancestors occur in the fossil record, both as ancestors of other extinct species, and ancestors of living and fossil lineages [15,17]. The challenge has always been to derive an acceptable method for reconciling the homology statements that characters represent, with the potential phylogenetic informativeness of fossil ages. Much of this debate has been considered within the parsimony framework of phylogenetic inference, but the application of likelihood-based models of character evolution that better accommodate homoplasy, provides a more appropriate framework in which to develop this debate further. The FBD model provides the basis for an objective approach to the identification of ancestors in the fossil record and, thus, for overcoming the traditional criticism of cladistic approaches to palaeontology—that in failing to observe absolute relationships, they artefactually inflate the perception of the gaps in the fossil record [18–20]. The failure to consider the possibility that fossil species might even be indirect ancestors of living lineages similarly serves to distance fossil minima from perceptions of clade age and so the recognition of fossil ancestors will serve to bring divergence time estimates into a closer approximation of fossil evidence. However, in the development of such methods, it is important that they control for non-uniform fossil preservations in stratigraphic sequence [9,39], as well as non-uniform losses of anatomical data in the process of decay and preservation that led to fossilization [83–85]. Many fossil species are compatible with ancestors, i.e. they do not exhibit autapomorphies [13], but only as an artefact of incomplete preservation. Thus, the accommodation of hypotheses of ancestry might lead to molecular clock estimates that achieve an entente with fossil evidence, but only by effectively concealing real gaps in the fossil record that are otherwise indicated by the existence of fossils that are siblings, rather than ancestors of living lineages.

## 5. Concluding remarks

Molecular clock methodology is undergoing a period of development unparalleled in the half century since the molecular clock hypothesis was first formulated. This has been brought about principally by the introduction of Bayesian inference, which provides a powerful framework for integrating different sources of information, with the uncertainties appropriately accommodated. In our perception at least, methods are diversifying, rather than new methods superseding established approaches. Indeed, there is now a broader palate of methods and approaches to divergence time estimation than there has been at any time in the past and these may be assembled in a combination that best suits the testing of the hypothesis at hand. Many of these components, like tip-calibration, the morphological clock and the FBD model, are at an early stage of development and current applications may not stand the test of time. Nevertheless, this palate of tools is already being assembled into a toolkit (e.g. [81]) that has the promise of developing into a fully integrative framework for calibrating the Tree of Life to geologic time, including all of its branches, living and dead. Many of the challenges that confront the development of molecular clock methodology exhibit striking parallels to long-standing debates in palaeontology, such as the role of time in topology estimation, the efficacy of attempts to identify direct and indirect ancestors among fossil taxa, as well as the impact on topology estimation of the non-uniform stratgraphic distribution and preservation of fossil species. Thus, methodological advances may be more readily achieved by learning from, rather than rehearsing, these debates. Rather than perpetuating controversy between molecular systematists and palaeontologists, in its middle age the molecular clock hypothesis looks set to serve as a nexus, dissolving the artificial barriers between these disciplines and their perceptions of evolutionary history.

## Data accessibility

There are no data associated with this study.
